# Research Progress of Ponticulus Posticus: A Narrative Literature Review

**DOI:** 10.3389/fsurg.2022.834551

**Published:** 2022-03-22

**Authors:** Xiaoyan Xu, Yuefeng Zhu, Xing Ding, Mengchen Yin, Wen Mo, Junming Ma

**Affiliations:** ^1^Shanghai University of Traditional Chinese Medicine, Shanghai, China; ^2^Department of Orthopaedics, Huadong Hospital, Fudan University, Shanghai, China; ^3^Department of Orthopaedics, Longhua Hospital, Shanghai University of Traditional Chinese Medicine, Shanghai, China

**Keywords:** ponticulus posticus (PP), research progress, narrative review, clinical presentation, surgical significance

## Abstract

**Study Design:**

Narrative review.

**Objective:**

The purpose of this review was to consolidate the current literature related to ponticulus posticus (PP) and to improve the systematic understanding of this anatomical variant of atlas among spine surgeons.

**Methods:**

Articles reviewed were searched in PubMed, Ovid MEDLINE, and Embase. All articles of any study design discussing on PP were considered for inclusion. Two independent authors read article titles and abstracts and included appropriate articles. The relevant articles were studied in full text.

**Results:**

A total of 113 literatures were reviewed and consolidated in this narrative review. These articles are roughly divided into the following five subcategories: (1) epidemiology, (2) pathology and anatomy, (3) clinical presentation, (4) surgical significance, and (5) radiographic examination.

**Conclusion:**

The PP is non-negligible with a high prevalence. The PP compresses the V3 segment of the artery, the suboccipital nerve, and the venous plexus, consequently contributing to the incidence of neurological pathologies. When a PP is observed or suspected on a lateral radiograph, we recommend that a computed tomography (CT) scan of a patient who is about to receive a C1 lateral mass screw (C1LMS) should be performed, which could determine a safe entry point and the right trajectory of screw insertion.

## Introduction

Ponticulus posticus (PP) is the meaning of “little posterior bridge” in Latin, which was a variation occurring on the atlas vertebra. It was defined as a bony bridge formed between the posterior portion of the superior articular process and the lateral portion of the upper margin of the posterior arch of the atlas, surrounding all or part of the vertebral artery (VA) (1). It was first detected on imaging incidentally and was reported in the dentistry, neurosurgery, and orthopedic spinal surgery literature (2).

Ponticulus posticus has not been a matter of concern for spine surgeons until an increasing number of epidemiology studies indicated its non-negligible morbidity. More published studies showed a close connection between PP and cervicogenic headache (CGH) (3). Surgical significance of PP in the insertion of screws into the lateral mass of the atlas was also reported (4). A practical, narrative review of PP was undertaken to address the following areas: (1) epidemiology, (2) pathology and anatomy, (3) clinical presentation, (4) surgical significance, and (5) radiographic examination. Not only did it provide an extensive systematic review of all recent studies, we would rather aim to provide an updated comprehensive synthesis of the current evidence to facilitate a cogent clinical understanding of PP, which could guide spine surgeons in the condition of cervical spine disorders combined with PP.

## Methods

A comprehensive literature search was performed on November 01, 2021 according to the Preferred Reporting Items for Systematic Review and Meta-Analyses (PRISMA) guidelines. Studies published from 1950 to 2021 were chosen through relevant PubMed, Ovid MEDLINE, and Embase searches to prioritize the largest and most recent studies. The Medical Subject headings and Boolean operators employed for this search were: “ponticulus posticus” or “posticus ponticus” or “foramen arcuate” or “foramen arcuale” or “foramen sagittale” or “foramen atlantoideum posterior” or “Kimmerle's anomaly” or “foramen retroarticulare superior” or “canalis vertebralis” or “retroarticular vertebral artery ring” or “retroarticular canal” or “retrocondylar vertebral artery”. Though no strict inclusion/exclusion criteria were used, preference was given to well-known, large, multi-institution databases that represented care across many centers, in addition to larger single-center studies. All articles about study design discussing about PP were considered for inclusion. Experimental or animal studies, non-English language studies, non-peer-reviewed studies, conference abstracts, paper, letter, and unpublished manuscripts were excluded. After an initial screen of abstracts and article titles, we obtained full-text articles of all potential studies. To perfect the research, two independent researchers reviewed and evaluated the included articles, respectively. Any different opinions were discussed until a consensus was reached. Since no human subjects were directly involved in this article; hence, an IRB statement was not needed.

## Results

### Literature Search

**Figure 1:**
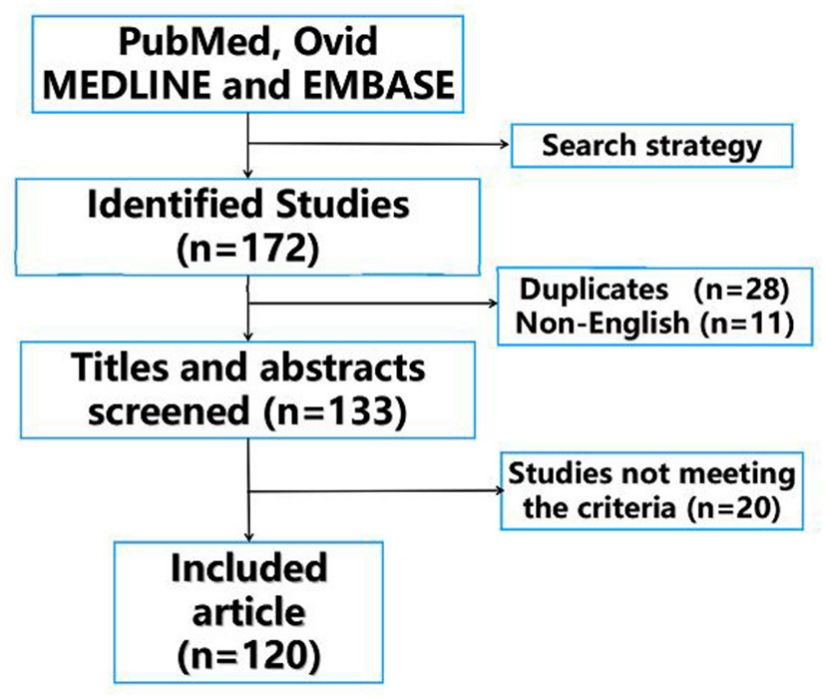
Flowchart of the identification, evaluation, and inclusion of studies in the review.

A total of 172 studies were identified from the initial search, of which 28 duplicates and 11 non-English language articles were removed. Titles and abstracts of the rest 133 studies were screened according to the predefined inclusion criteria, and 20 studies were excluded. In total, 113 articles were critically reviewed and consolidated for this literature review (Figure 1).

### Epidemiology

Ponticulus posticus is a normal anatomical variant of atlas vertebrae (C1), and its prevalence in population has been the focus in PP studies. In the current study, we updated the studies on the prevalence of PP in various areas of the world, and a total of 58 published studies were included in the narrative review (Table 1). According to the review, we found that the total prevalence of these studies was 5–55.7%, and there were some regional differences in the prevalence. The prevalence in East Asia was 6.2–19.0%, Europe was 14.3–34.7%, North America was 5–45.5%, and India was 10.9–37.8%, of which East Asia had the lowest incidence. These differences could be attributed to the differences in the different ethnic groups all over the world. Some scholars argued that the degenerative changes may be the cause of PP, and prevalence increases with age due to calcification, but there was no definitive evidence for association between the age and the prevalence of PP. Several recent studies did not find a statistically significant association between the age and the presence of PP (7–9). With regard to sex bias, scholars hold different views. In studies conducted by Takaaki, Paraskevas, Hong, and Saleh, the frequency of PP was higher in men (9–12). In contrast, the studies conducted by Schilling et al. (2010) and Tambawala et al. reported female predilection for this anomaly (13, 14). More studies showed that there was no statistically significant association between gender of the patient and the presence of PP (7, 8, 15, 16). The currently available literature was inconclusive in this aspect. According to Pekala's meta-analysis of 55,985 subjects, the total prevalence of the incomplete PP was 13.6%, which was higher than the complete one (9.1%) (3). However, the meta-analysis performed by Elliott and Tanweer (17) found complete PP in 9.3% of patients and incomplete PP in 8.7% of patients. The difference of study results may be attributed to the methods employed, and we could not reach a definitive conclusion in this aspect. In terms of laterality, the study conducted by Saleh et al. (9) indicated that the left sided arch has a higher rate of PP than the right one (84.7 vs. 89.2%), which was consistent with the findings made in the study of Elliott and Tanweer (17).

**Table 1:** Review of the literatures on prevalence of PP.

	**Author**	**Year**	**Sample**	**Population**	**PP (%)**
1	S Selby	1955	306	USA	27.1%
2	Pyo J	1959	300	USA	12.7%
3	Kendrick GS	1963	353	USA	15.8%
4	Radojevic S	1964	105	Sweden	14.3%
5	Saunders SR	1978	592	Canada	29.2%
6	Farman AG	1979	220	South Africa	8.0%
7	Gupta SC	1979	123	India	18.7%
8	Takaaki M	1979	307	Japan	9.1%
9	Taitz C	1986	672	Multiple continents	33.8%
10	Ruprecht A	1988	419	Saudi Arabia	32.9%
11	Sun JY	1990	923	China	7.4%
12	Le Mino	1992	500	France	14.2%
13	Stubbs	1992	1,000	USA	18.7%
14	Dhall U	1993	148	India	37.8%
15	Mitchell J	1998	1,354	South Africa	9.8%
16	Wight S	1999	895	Scotland	18.0%
17	Cederberg RA	2000	255	North America	11.0%
18	Hasan M	2001	350	North India	6.6%
19	Manjunath KY	2001	60	South India	11.7%
20	Wysocki J	2003	95	Poland	31.6%
21	Kavakli A	2004	86	Turkey	22.1%
22	Unur E	2004	351	Turkey	5.1%
23	Beck RW	2004	847	New Zeland	13.6%
24	Cakmak O	2005	476	Turkey	13.7%
25	Young JP	2005	464	USA	15.5%
26	Paraskevas G	2005	176	Greece	34.7%
27	Senoglu M	2006	338	Turkey	15.2%
28	Lee MJ	2006	709	USA	26.9%
27	Krishnamurthy A	2007	1044	India	13.8%
28	Tubbs RS	2007	60	USA	5.0%
29	Kim KH	2007	537	Korea	19.0%
30	Gupta T	2008	55	India	10.9%
31	Kobayashi Y	2008	50	Japan	10.0%
32	Simsek S	2008	158	Turkey	9.5%
33	Hong JT	2008	1013	Korea	15.6%
34	Cho	2009	355	Korea	11.8%
35	Karau PB	2010	102	Kenya	28.4%
36	Kuhta P	2010	246	USA	45.5%
37	Schilling J	2010	436	USA	19.3%
38	Yeom JS	2012	52	Korea	17.3%
39	Carvalho MF	2012	30	Brasil	40%
40	Baeesa SS	2012	453	Saudi Arabia	47.9%
41	Bayrakdar IS	2014	730	Turkey	17.4%
42	Perez IE	2014	1056	Peruvia	19.8%
43	Geist JR	2014	576	USA	26.2%
44	Wakao N	2014	387	Japan	6.2%
45	Chen CH	2015	500	Taiwan	7.0%
46	Gibelli D	2015	221	Italy	16.7%
47	Sekerci AE	2015	698	Turkey	36.8%
48	Tambawala SS	2017	500	Indian	15.8%
49	Giri J	2017	414	Nepal	35.7%
50	Cirpan S	2017	81	Turkey	16.1%
51	Buyuk SK	2017	374	Nepal	43.0%
52	Song MS	2017	2628	Korea	7.1%
53	Sanchis-Gimeno JA	2018	300	Spain	20.3%
54	Bayrakdar IS	2018	181	Turkey	36.5%
55	Saleh A	2018	2917	USA	22.5%
56	Tripodi D	2019	524	Italy	28.2%
57	Evirgen S	2020	440	Turkey	55.7%
58	Arada CY	2021	108	Thailand	10.3%

### Pathology and Anatomy

Ponticulus posticus is an osseous prominence formed in place of a sulcus for the VA on the posterior arch of the atlas. The atlas with a particular anatomy is composed with a short anterior arch and a longer posterior arch, which is a ring-shaped structure without vertebral body. The vertebral artery groove (VAG) is on the superior surface of the posterior arch (18, 19). PP is an aperture formed by the presence of a bony bridge on the VAG, which is placed posteriorly in relation to the anterior surface, and when the bridge is placed laterally, it is called ponticulus lateralis (PL) – a rare type of PP.

The prevalence of PL was reported to be 1.8–3.8% lower than PP (20–23). PL is difficult to be identified from anteroposterior and lateral radiographs and was rarely reported in previous literature as a result. The V3 segment of the VA travels in the VAG, which is covered by a bony ridge with the presence of PP. According to our literature review, the prevailing view was that PP compresses the V3 segment of the VA and causes alternations of the blood flow within the VAs that are ultimately responsible for a range of symptoms such as migraine and CGH. More than 50% of head rotation occurred at the atlantoaxial joint. With additional compression caused by PP, VA is more susceptible to injury when subjected to compression and extension (24). According to the study of atlas vertebrae from the population of northern Greece by Paraskevas et al., there was a high incidence of the coexistence of PP with retrotransverse forame. (11). It reported that the blood flow was directed into the small vein connecting the atlanto-occipital and the atlanto-axoidian venous sinus due to the compression of the vertebral veins in PP. This study also found that 93.5% cases of PP were accompanied by deeply excavated contralateral groove of the VA, which could be interpreted as evidence that, due to VA compression in the canal, the contralateral VA was dilated, causing an increase in the depth of the corresponding groove. In the study of cadaver conducted by Tubbs et al., all specimens with PP were also found to have gross compression of the VA as it traveled through the PP (25).

### Clinical Presentation

From an anatomical point of view, PP compresses the V3 segment of the artery, the suboccipital nerve, and the venous plexus, consequently contributing to the incidence of neurological pathologies such as vertigo and migraine (26). Pekala conducted a meta-analysis in 2018, finding a significant association between PP and headaches (3). Besides, the probability of complete PP resulting in headaches was higher than the incomplete ones, which in turn had a higher probability of headache compared to patients without PP. This result indicated the importance of PP in the etiology of headaches, which was supported by multiple prior studies (14, 27–29). Except for headaches, PP could cause a range of symptoms including retro-orbital pain, vasomotor disturbance of the face and recurrent disturbances of vision, swallowing, and phonation—the Barre–Lieou syndrome caused by alteration of the blood flow within the vertebral arteries, and an associated disturbance of the periarterial nerve plexus (30).

The study by Pearce (2004) introduced the ponticulus resection to treat the Barre–Lieou syndrome caused by PP (30). The patients who had surgical resection of PP during the last 10 years were reviewed and satisfactory surgical outcomes were found. However, we could not find any studies on this topic in recent years. We conjectured that few patients with the Barre–Lieou syndrome were serious enough to require surgical resection.

In addition to neurological pathologies, PP is associated with oral and maxillofacial disorders. This may be attributed to the activity of the neural crest as the common embryonic origin of the neck and shoulder skeletal development and the origin of development of tooth and midface skeletal fields (31, 32). According to the study conducted by Dadgar et al., the presence of the PP correlated with the presence of palatally displaced canines (PDC) significantly and positively (33). The study by Leonardi et al. (31) reached a converging conclusion in which 34.2% of patients with PDC showed PP as opposed to the group of normal population (20%) (34). Bayrakdar et al. found that there was a significant association between PP and cleft lip (35). In this study, the incidence of PP in the cleft-palate group was 22.2% compared to 9% in normal group.

### Radiographic Examination

**Figure 2:**
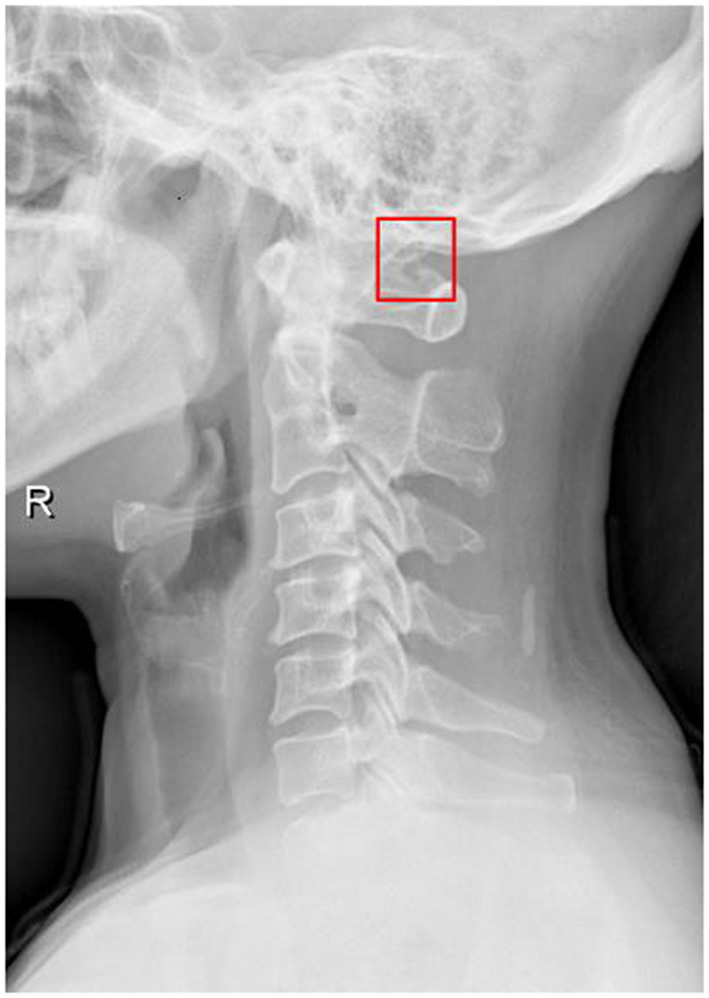
A 44-year-old female with migraine and normal neurologic examination. Lateral radiograph showed the right-sided partial ponticulus posticus, an anomalous bony bridge formed from the superior articulating surface of the atlas but not fused to the posterior arch of the atlas.

We noticed that there were several methodologies to identify the PP in previous studies including cadaveric studies, lateral radiographs, and computed tomography (CT) scans. In our study, we found that the prevalence of PP in different studies was different, which may be contributed to the methodologies. In the radiographic examination, lateral radiographs could not identify the laterality, completeness, and sometimes even the presence of PP. In the study by Kim et al., the prevalence of PP was 26% based on the CT scans, which was only 14% in lateral radiographs (36). The difference was significant and meant that a substantial proportion of patients with PP were missed on lateral radiographs. The CT scan was still a reliable method when PP was combined with other anatomical variant. Figure 2 shows a typical case. Elgafy et al. reported a special CT finding of ipsilateral PP and high-riding VA, which were found only in 5 patients out of 100 cases (37).

Radiographically, the most common classification of PP was based on the completeness of the bony bridge: none, complete, and incomplete. None type: there was no formed bony bridge; complete type: a complete bony ring was formed; and incomplete type: some portions of the bony ring were defective. However, this traditional classification neglected the laterality of PP, and there was a novel classification system introduced by Saleh et al. (9). This classification consisted of a two-letter designation for each patient, including either A, B, or C (A means no PP; B means incomplete PP; and C means complete PP). The first letter described the right-sided posterior arch, and the second letter described the left one. This classification system included 9 potential subtypes for all patients: AA, BB, CC, AB, AC, BA, BC, CA, and CB. However, we could not find a classification that combines clinical symptoms with imaging findings.

### Surgical Significance

The C1 lateral mass screw (C1LMS) insertion was firstly reported by Goel and Laheri in 1994, which revolutionized the treatment of atlantoaxial instability (38). PP has gained increasing attention in recent years, and the literature has increased correspondingly as C1LMS has become increasingly popular. When the methods of inserting the C1LMS is through the posterior arch into the lateral mass, PP may be mistaken for a thickened posterior arch and may mislead the surgeon to drill the borehole too superiorly, which could cause iatrogenic injury to the V3 segment of the artery. Zhang et al. successfully inserted C1LMS in 11 patients with PP by performing preoperative three-dimensional CT reconstructive imaging, which contributed to choose an appropriate entry point and a right trajectory of screw insertion (39). Arslan et al. developed cervical column 3D models for 200 patients, of which 29 were with PP, and evaluated 3D models of both normal and PP cases (6). They found that the VA in PP cases was clearly narrower than that in normal cases, and the safe distance between lateral mass screw fixation and the bony bridge was 4 mm.

The conventional C1LMSs have been accepted as more stable approaches to avoid VA injury compared with C1LMSs inserted *via* the posterior arch because the screws are placed farther from the VAG. However, the study by Song et al. indicated that the latter had some anatomical feasibility and advantage with the relatively sufficient VAG height (40). In addition, the lower margin of the C1 arch could determine an appropriate entry point. The disadvantage of the conventional C1LMSs included more venous bleeding, less biomechanical stability, and postoperative C2 nerve dysfunction.

In the study conducted by Yeom et al., 9 patients with PP received C1LMS, and 3 of whom received resection of the ponticulus before the screw insertion due to wide PP and deep VAG (41). Although VA injury was not reported in this study, we did not advocate this radical surgery strategy. Notably, Lee et al. reported the notching technique (lateral mass screws inserted partially through the posterior arch), which modified the entry point to make the screw remote from the greater occipital nerve and was possible in the vast majority of patients (42).

## Conclusion

Considering different methodologies and regional differences, the prevalence of PP is inconsistent. However, one point is certain, PP is non-negligible with a high prevalence. PP compresses the V3 segment of the artery, the suboccipital nerve, and the venous plexus, consequently contributing to the incidence of neurological pathologies. When a PP is observed or suspected on a lateral radiograph, we recommend that a CT scan of a patient who is about to receive a C1LMS should be performed, which could determine a safe entry point and a right trajectory of screw insertion. The insertion of C1LMSs *via* the posterior arch was applicable in the majority of cases, and the notching technique might be considered as necessary. Conventional C1LMSs should not be recommended due to the surgical risk and the postoperative complications.

## Author Contributions

MY, XX, and YZ: conceptualization, methodology, formal analysis, and writing-original draft. WM: resources and data curation. XD and JM: supervision and writing-reviewing and editing. All authors read and approved the final manuscript.

## Conflict of Interest

The authors declare that the research was conducted in the absence of any commercial or financial relationships that could be construed as a potential conflict of interest.

## Publisher's Note

All claims expressed in this article are solely those of the authors and do not necessarily represent those of their affiliated organizations, or those of the publisher, the editors and the reviewers. Any product that may be evaluated in this article, or claim that may be made by its manufacturer, is not guaranteed or endorsed by the publisher.

## Abbreviations

PP: ponticulus posticus
C1LMS: C1 lateral mass screw
CGH: cervicogenic headache
PDC: palatally displaced canines
PL: ponticulus lateralis
VAG: vertebral artery groove.
